# 1-(2-Methyl­benz­yl)-1*H*-indole-3-carbaldehyde

**DOI:** 10.1107/S1600536812010306

**Published:** 2012-03-24

**Authors:** Yang Wu, Wen Ren, Qiang Wang, Gu He

**Affiliations:** a State Key Laboratory of Biotherapy and Cancer Center, West China Hospital, West China Medical School, Sichuan University, Chengdu 610041, People’s Republic of China; b West China School of Pharmacy, Sichuan University, Chengdu 610041, People’s Republic of China; c State Key Laboratory of Biotherapy and Cancer Center, West China Hospital, West China Medical School, Sichuan University, Chengdu 610041, People’s Republic of China,

## Abstract

In the title compound, C_17_H_15_NO, the benzene ring and the indole system are almost perpendicular, making a dihedral angle of 87.82 (6)°. The crystal packing is stabilized by C—H⋯O and π–π stacking inter­actions with centroid–centroid distances of 3.592 (4) Å between the pyrrole and the benzene rings in the indole systems of neighboring mol­ecules.

## Related literature

For general background to the chemistry and anti-inflammatory activity of carboxylic acid derivatives, see: Andreani *et al.* (1994 ▶).
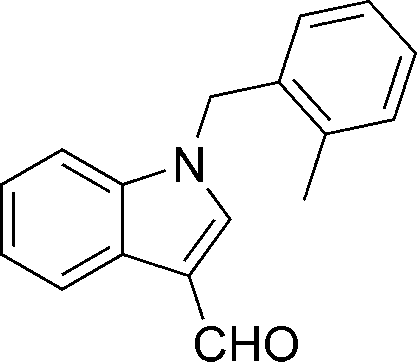


## Experimental

### 

#### Crystal data

C_17_H_15_NO
*M* *_r_* = 249.30
Monoclinic, 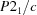

*a* = 10.5251 (3) Å
*b* = 15.4352 (5) Å
*c* = 8.2335 (2) Å
β = 99.214 (3)°
*V* = 1320.33 (7) Å^3^
*Z* = 4
Mo *K*α radiation
μ = 0.08 mm^−1^
*T* = 293 K
0.28 × 0.25 × 0.20 mm

#### Data collection

Agilent Xcalibur Eos diffractometer
Absorption correction: multi-scan (*CrysAlis PRO*; Agilent, 2011 ▶) *T*_min_ = 0.981, *T*_max_ = 1.000
5468 measured reflections
2699 independent reflections
1928 reflections with *I* > 2σ(*I*)
*R*_int_ = 0.016

#### Refinement

*R*[*F* ^2^ > 2σ(*F* ^2^)] = 0.047
*wR*(*F* ^2^) = 0.117
*S* = 1.04
2699 reflections
173 parameters
H-atom parameters constrained
Δρ_max_ = 0.13 e Å^−3^
Δρ_min_ = −0.17 e Å^−3^

### 

Data collection: *CrysAlis PRO* (Agilent, 2011 ▶); cell refinement: *CrysAlis PRO*; data reduction: *CrysAlis PRO*; program(s) used to solve structure: *SHELXS97* (Sheldrick, 2008 ▶); program(s) used to refine structure: *SHELXL97* (Sheldrick, 2008 ▶); molecular graphics: *OLEX2* (Dolomanov, 2009 ▶); software used to prepare material for publication: *OLEX2*.

## Supplementary Material

Crystal structure: contains datablock(s) I, global. DOI: 10.1107/S1600536812010306/bt5840sup1.cif

Structure factors: contains datablock(s) I. DOI: 10.1107/S1600536812010306/bt5840Isup2.hkl

Supplementary material file. DOI: 10.1107/S1600536812010306/bt5840Isup3.cml

Additional supplementary materials:  crystallographic information; 3D view; checkCIF report

## Figures and Tables

**Table 1:** Hydrogen-bond geometry (Å, °)

*D*—H⋯*A*	*D*—H	H⋯*A*	*D*⋯*A*	*D*—H⋯*A*
C16—H16⋯O1^i^	0.93	2.56	3.418 (2)	154

Symmetry code: (i) 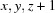.

**Fig. 1.**
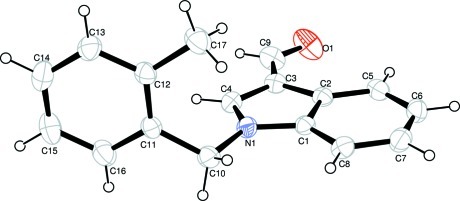
The molecular structure of the title compound, with displacement ellipsoids drawn at the 30% probability level.

**Fig. 2.**
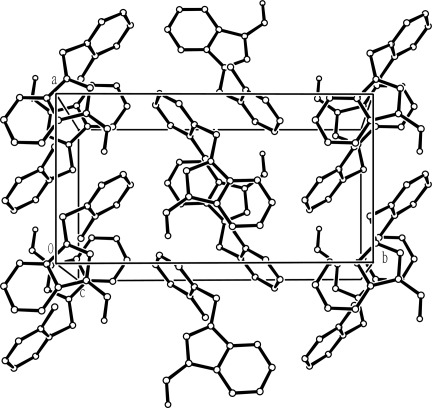
A packing diagram of the title compound.

## supplementary crystallographic information

### Comment

1-(2-Methyl-benzyl)-1*H*-indole-3-carbaldehyde is of great importance owing to its wide biological properties (Andreani *et al.*, 1994). The title compound is one of the key intermediates in our synthetic investigations of antibacterial drugs. We report here its crystal structure.

In the title compound, C_17_H_15_NO, as shown in Fig 1, the benzene ring with the methyl group in *ortho* position and the indole system are almost perpendicular, making dihedral angle of 87.82 (6)°. A combination of intermolecular C-H···O and π–π packing interaction plays an important role in the connection of neighbouring molecules. The centroid-centroid distance between the pyrrole ring and the benzene ring in the indole system of the neighbouring molecule is 3.592 (4) Å (symmetry operator: -x, -y, -z).

### Experimental

1*H*-indole-3-carbaldehyde(14 mmol) was dissolved in dry DMF(25 ml)and treated portionwise,under stirring, with 20 mmol NaH. The mixture was stirred at room temperature for 10 min and treated with 20 mmol of 1-chloromethyl-2-methyl-benzene. After 1 h at 90°C under stirring, the mixture was poured onto ice. The resulting precipitate was collected by filtration and crystallized from ethanol with a yield of 70%. Crystals suitable for X-ray analysis were obtained by slow evaporation from a solution of dichloromethane.

### Refinement

All H atoms were positioned geometrically and refined using a riding model, with C—H = 0.93–0.97 Å and with *U*_iso_(H) = 1.2 or 1.5*U*_eq_(C) for methyl H atoms.

### Crystal data

**Table:**

C_17_H_15_NO	*F*(000) = 528
*M**_r_* = 249.30	*D*_x_ = 1.254 Mg m^−^^3^
Monoclinic, *P*2_1_/*c*	Mo *K*α radiation, λ = 0.7107 Å
*a* = 10.5251 (3) Å	Cell parameters from 1937 reflections
*b* = 15.4352 (5) Å	θ = 3.2–29.1°
*c* = 8.2335 (2) Å	µ = 0.08 mm^−^^1^
β = 99.214 (3)°	*T* = 293 K
*V* = 1320.33 (7) Å^3^	Block, colorless
*Z* = 4	0.28 × 0.25 × 0.20 mm

### Data collection

**Table:**

Agilent Xcalibur Eos diffractometer	2699 independent reflections
Radiation source: fine-focus sealed tube	1928 reflections with *I* > 2σ(*I*)
Graphite monochromator	*R*_int_ = 0.016
Detector resolution: 16.0874 pixels mm^-1^	θ_max_ = 26.4°, θ_min_ = 3.2°
ω scans	*h* = −11→13
Absorption correction: multi-scan (*CrysAlis PRO*; Agilent, 2011)	*k* = −11→19
*T*_min_ = 0.981, *T*_max_ = 1.000	*l* = −10→4
5468 measured reflections	

### Refinement

**Table:**

Refinement on *F*^2^	Primary atom site location: structure-invariant direct methods
Least-squares matrix: full	Secondary atom site location: difference Fourier map
*R*[*F*^2^ > 2σ(*F*^2^)] = 0.047	Hydrogen site location: inferred from neighbouring sites
*wR*(*F*^2^) = 0.117	H-atom parameters constrained
*S* = 1.04	*w* = 1/[σ^2^(*F*_o_^2^) + (0.0449*P*)^2^ + 0.2065*P*] where *P* = (*F*_o_^2^ + 2*F*_c_^2^)/3
2699 reflections	(Δ/σ)_max_ < 0.001
173 parameters	Δρ_max_ = 0.13 e Å^−^^3^
0 restraints	Δρ_min_ = −0.17 e Å^−^^3^

### Special details

**Table:**

Geometry. All e.s.d.'s (except the e.s.d. in the dihedral angle between two l.s. planes) are estimated using the full covariance matrix. The cell e.s.d.'s are taken into account individually in the estimation of e.s.d.'s in distances, angles and torsion angles; correlations between e.s.d.'s in cell parameters are only used when they are defined by crystal symmetry. An approximate (isotropic) treatment of cell e.s.d.'s is used for estimating e.s.d.'s involving l.s. planes.
Refinement. Refinement of *F*^2^ against ALL reflections. The weighted *R*-factor *wR* and goodness of fit *S* are based on *F*^2^, conventional *R*-factors *R* are based on *F*, with *F* set to zero for negative *F*^2^. The threshold expression of *F*^2^ > σ(*F*^2^) is used only for calculating *R*-factors(gt) *etc*. and is not relevant to the choice of reflections for refinement. *R*-factors based on *F*^2^ are statistically about twice as large as those based on *F*, and *R*- factors based on ALL data will be even larger.

### Fractional atomic coordinates and isotropic or equivalent isotropic displacement parameters (Å^2^)

**Table:**

	*x*	*y*	*z*	*U*_iso_*/*U*_eq_	
O1	0.02058 (15)	0.14964 (11)	−0.33644 (17)	0.0895 (5)	
N1	0.21128 (12)	0.01830 (9)	0.14071 (15)	0.0439 (3)	
C1	0.18887 (13)	−0.04418 (11)	0.01839 (19)	0.0404 (4)	
C2	0.13295 (13)	−0.00278 (11)	−0.12762 (19)	0.0415 (4)	
C3	0.12187 (14)	0.08699 (11)	−0.0886 (2)	0.0457 (4)	
C4	0.17018 (15)	0.09542 (11)	0.0760 (2)	0.0475 (4)	
H4	0.1739	0.1472	0.1344	0.057*	
C5	0.10025 (15)	−0.05289 (13)	−0.2699 (2)	0.0506 (4)	
H5	0.0639	−0.0273	−0.3687	0.061*	
C6	0.12281 (16)	−0.14068 (13)	−0.2611 (2)	0.0573 (5)	
H6	0.1010	−0.1744	−0.3549	0.069*	
C7	0.17749 (16)	−0.17975 (12)	−0.1149 (2)	0.0567 (5)	
H7	0.1910	−0.2393	−0.1126	0.068*	
C8	0.21221 (15)	−0.13246 (11)	0.0266 (2)	0.0503 (4)	
H8	0.2499	−0.1587	0.1242	0.060*	
C9	0.06793 (18)	0.15646 (14)	−0.1918 (3)	0.0642 (5)	
H9	0.0690	0.2113	−0.1449	0.077*	
C10	0.26384 (17)	0.00142 (12)	0.31387 (19)	0.0536 (5)	
H10A	0.3153	−0.0510	0.3210	0.064*	
H10B	0.1933	−0.0082	0.3745	0.064*	
C11	0.34564 (15)	0.07493 (11)	0.39236 (19)	0.0455 (4)	
C12	0.46514 (15)	0.09284 (11)	0.34816 (19)	0.0448 (4)	
C13	0.53745 (17)	0.15965 (12)	0.4283 (2)	0.0565 (5)	
H13	0.6176	0.1721	0.4002	0.068*	
C14	0.4941 (2)	0.20792 (14)	0.5479 (2)	0.0679 (6)	
H14	0.5445	0.2525	0.5997	0.082*	
C15	0.3765 (2)	0.19041 (15)	0.5909 (3)	0.0744 (6)	
H15	0.3467	0.2228	0.6722	0.089*	
C16	0.30237 (18)	0.12415 (14)	0.5125 (2)	0.0637 (5)	
H16	0.2221	0.1125	0.5411	0.076*	
C17	0.51550 (17)	0.04237 (15)	0.2160 (2)	0.0666 (6)	
H17A	0.5075	−0.0185	0.2360	0.100*	
H17B	0.6044	0.0565	0.2168	0.100*	
H17C	0.4667	0.0568	0.1108	0.100*	

### Atomic displacement parameters (Å^2^)

**Table:**

	*U*^11^	*U*^22^	*U*^33^	*U*^12^	*U*^13^	*U*^23^
O1	0.1027 (11)	0.1071 (13)	0.0562 (9)	0.0489 (10)	0.0050 (8)	0.0189 (9)
N1	0.0436 (7)	0.0468 (8)	0.0395 (7)	−0.0044 (6)	0.0014 (6)	0.0004 (7)
C1	0.0344 (8)	0.0448 (10)	0.0419 (9)	−0.0048 (7)	0.0059 (6)	−0.0003 (8)
C2	0.0307 (7)	0.0519 (10)	0.0418 (9)	−0.0009 (7)	0.0059 (6)	0.0016 (8)
C3	0.0377 (8)	0.0520 (11)	0.0471 (9)	0.0042 (7)	0.0054 (7)	0.0049 (8)
C4	0.0461 (9)	0.0446 (10)	0.0515 (10)	−0.0020 (7)	0.0070 (8)	−0.0009 (8)
C5	0.0386 (8)	0.0719 (13)	0.0404 (9)	−0.0027 (8)	0.0031 (7)	−0.0013 (9)
C6	0.0509 (10)	0.0663 (13)	0.0550 (11)	−0.0111 (9)	0.0098 (8)	−0.0169 (10)
C7	0.0570 (10)	0.0472 (11)	0.0663 (12)	−0.0060 (8)	0.0109 (9)	−0.0063 (9)
C8	0.0495 (9)	0.0482 (11)	0.0523 (10)	−0.0042 (8)	0.0052 (8)	0.0054 (9)
C9	0.0626 (12)	0.0655 (13)	0.0659 (13)	0.0204 (10)	0.0145 (10)	0.0106 (11)
C10	0.0586 (10)	0.0626 (12)	0.0381 (9)	−0.0110 (9)	0.0031 (8)	0.0043 (8)
C11	0.0486 (9)	0.0514 (10)	0.0348 (8)	−0.0002 (8)	0.0020 (7)	0.0014 (8)
C12	0.0459 (9)	0.0520 (10)	0.0345 (8)	0.0019 (8)	0.0004 (7)	0.0041 (8)
C13	0.0511 (10)	0.0644 (12)	0.0505 (10)	−0.0082 (9)	−0.0028 (8)	0.0026 (10)
C14	0.0765 (13)	0.0592 (13)	0.0622 (12)	−0.0081 (11)	−0.0073 (11)	−0.0119 (11)
C15	0.0856 (15)	0.0753 (15)	0.0612 (13)	0.0110 (12)	0.0082 (11)	−0.0251 (11)
C16	0.0579 (11)	0.0806 (15)	0.0541 (11)	0.0017 (10)	0.0133 (9)	−0.0112 (10)
C17	0.0572 (11)	0.0863 (16)	0.0584 (12)	0.0029 (10)	0.0161 (9)	−0.0093 (11)

### Geometric parameters (Å, º)

**Table:**

O1—C9	1.220 (2)	C9—H9	0.9300
N1—C1	1.387 (2)	C10—H10A	0.9700
N1—C4	1.347 (2)	C10—H10B	0.9700
N1—C10	1.4677 (19)	C10—C11	1.506 (2)
C1—C2	1.405 (2)	C11—C12	1.392 (2)
C1—C8	1.384 (2)	C11—C16	1.381 (2)
C2—C3	1.431 (2)	C12—C13	1.385 (2)
C2—C5	1.400 (2)	C12—C17	1.502 (2)
C3—C4	1.375 (2)	C13—H13	0.9300
C3—C9	1.428 (2)	C13—C14	1.370 (3)
C4—H4	0.9300	C14—H14	0.9300
C5—H5	0.9300	C14—C15	1.367 (3)
C5—C6	1.376 (3)	C15—H15	0.9300
C6—H6	0.9300	C15—C16	1.382 (3)
C6—C7	1.386 (3)	C16—H16	0.9300
C7—H7	0.9300	C17—H17A	0.9600
C7—C8	1.374 (2)	C17—H17B	0.9600
C8—H8	0.9300	C17—H17C	0.9600
			
C1—N1—C10	125.15 (14)	N1—C10—H10A	109.1
C4—N1—C1	108.69 (13)	N1—C10—H10B	109.1
C4—N1—C10	126.06 (14)	N1—C10—C11	112.57 (14)
N1—C1—C2	107.72 (14)	H10A—C10—H10B	107.8
C8—C1—N1	129.84 (15)	C11—C10—H10A	109.1
C8—C1—C2	122.44 (15)	C11—C10—H10B	109.1
C1—C2—C3	106.65 (14)	C12—C11—C10	121.05 (15)
C5—C2—C1	118.56 (16)	C16—C11—C10	119.37 (16)
C5—C2—C3	134.79 (16)	C16—C11—C12	119.57 (16)
C4—C3—C2	106.38 (14)	C11—C12—C17	121.66 (15)
C4—C3—C9	124.34 (17)	C13—C12—C11	118.30 (15)
C9—C3—C2	129.24 (16)	C13—C12—C17	120.04 (16)
N1—C4—C3	110.55 (15)	C12—C13—H13	119.1
N1—C4—H4	124.7	C14—C13—C12	121.77 (17)
C3—C4—H4	124.7	C14—C13—H13	119.1
C2—C5—H5	120.6	C13—C14—H14	120.1
C6—C5—C2	118.87 (16)	C15—C14—C13	119.88 (18)
C6—C5—H5	120.6	C15—C14—H14	120.1
C5—C6—H6	119.4	C14—C15—H15	120.3
C5—C6—C7	121.23 (17)	C14—C15—C16	119.49 (18)
C7—C6—H6	119.4	C16—C15—H15	120.3
C6—C7—H7	119.3	C11—C16—C15	121.00 (18)
C8—C7—C6	121.48 (18)	C11—C16—H16	119.5
C8—C7—H7	119.3	C15—C16—H16	119.5
C1—C8—H8	121.3	C12—C17—H17A	109.5
C7—C8—C1	117.41 (16)	C12—C17—H17B	109.5
C7—C8—H8	121.3	C12—C17—H17C	109.5
O1—C9—C3	125.3 (2)	H17A—C17—H17B	109.5
O1—C9—H9	117.4	H17A—C17—H17C	109.5
C3—C9—H9	117.4	H17B—C17—H17C	109.5
			
N1—C1—C2—C3	−0.28 (16)	C5—C2—C3—C9	1.2 (3)
N1—C1—C2—C5	−179.55 (13)	C5—C6—C7—C8	−0.5 (3)
N1—C1—C8—C7	178.63 (15)	C6—C7—C8—C1	0.9 (2)
N1—C10—C11—C12	−69.9 (2)	C8—C1—C2—C3	179.02 (14)
N1—C10—C11—C16	111.41 (18)	C8—C1—C2—C5	−0.3 (2)
C1—N1—C4—C3	−0.86 (18)	C9—C3—C4—N1	178.46 (15)
C1—N1—C10—C11	147.04 (15)	C10—N1—C1—C2	177.28 (13)
C1—C2—C3—C4	−0.23 (16)	C10—N1—C1—C8	−1.9 (2)
C1—C2—C3—C9	−177.87 (16)	C10—N1—C4—C3	−177.41 (13)
C1—C2—C5—C6	0.6 (2)	C10—C11—C12—C13	−178.10 (15)
C2—C1—C8—C7	−0.5 (2)	C10—C11—C12—C17	2.5 (2)
C2—C3—C4—N1	0.67 (17)	C10—C11—C16—C15	178.00 (18)
C2—C3—C9—O1	0.0 (3)	C11—C12—C13—C14	−0.3 (3)
C2—C5—C6—C7	−0.3 (2)	C12—C11—C16—C15	−0.7 (3)
C3—C2—C5—C6	−178.37 (16)	C12—C13—C14—C15	0.1 (3)
C4—N1—C1—C2	0.69 (16)	C13—C14—C15—C16	−0.2 (3)
C4—N1—C1—C8	−178.54 (15)	C14—C15—C16—C11	0.5 (3)
C4—N1—C10—C11	−37.0 (2)	C16—C11—C12—C13	0.5 (2)
C4—C3—C9—O1	−177.28 (17)	C16—C11—C12—C17	−178.81 (16)
C5—C2—C3—C4	178.87 (16)	C17—C12—C13—C14	179.10 (17)

### Hydrogen-bond geometry (Å, º)

**Table:**

*D*—H···*A*	*D*—H	H···*A*	*D*···*A*	*D*—H···*A*
C16—H16···O1^i^	0.93	2.56	3.418 (2)	154

Symmetry code: (i) *x*, *y*, *z*+1.
